# ANKS4B Restricts Replication of Zika Virus by Downregulating the Autophagy

**DOI:** 10.3389/fmicb.2020.01745

**Published:** 2020-07-22

**Authors:** Quanshi Lin, Shili Zhou, Yanxia Huang, Zhiting Huo, Cancan Chen, Xin Luo, Junfang He, Chao Liu, Ping Zhang

**Affiliations:** ^1^Key Laboratory of Tropical Disease Control, Zhongshan School of Medicine, Sun Yat-sen University, Ministry of Education, Guangzhou, China; ^2^Department of Immunology, Zhongshan School of Medicine, Sun Yat-sen University, Guangzhou, China; ^3^Department of Pathology, The First Affiliated Hospital of Sun Yat-sen University, Guangzhou, China; ^4^Department of Microbiology, Zhongshan School of Medicine, Sun Yat-sen University, Guangzhou, China

**Keywords:** Zika virus, ANKS4B, replication, autophagy, restriction factor

## Abstract

Infection of Zika virus (ZIKV) has become a severe threaten to global health while no specific drug is available. In this study, we explored the relationship between ZIKV and a cellular protein, ankyrin repeat and sterile motif domain containing 4b (ANKS4B). Our data revealed that the expression of *ANKS4B* in cultured cells and in neonatal mice was downregulated by ZIKV infection. The reduction of *ANKS4B* upon ZIKV infection was caused by decrease of two hepatocyte nuclear factors *HNF1α* and *HNF4α*. Through CRISPR/Cas9 gene editing system, we generated two ANKS4B knockout (KO) cell clones in A549 and Huh7 cells respectively. In the ANKS4B-KO cells, the viral replication levels including viral RNA, protein, and titer were significantly enhanced, which was reversed by *trans*-complementation of ANKS4B. ANKS4B did not affect the viral entry step, but impaired the autophagy induced by ZIKV infection. Furthermore, our data showed that inhibition of autophagy led to similar replication levels of ZIKV in ANKS4B-sufficient and ANKS4B-deficient cells, suggesting the antiviral effect of ANKS4B relied on its modulation on the autophagy. Therefore, our work identified ANKS4B as a new restriction factor of ZIKV.

## Introduction

Zika virus (ZIKV) is a re-emerging arbovirus in the genus Flavivirus of the family *Flaviviridae* (Musso and Gubler, 2016). ZIKV infection may be asymptomatic and symptomatic. The symptoms are usually mild and similar to other infectious diseases such as dengue fever, including fever, rash, arthralgia, and conjunctivitis (Petersen et al., 2016; Pierson and Diamond, 2018; Moutailler et al., 2019). However, ZIKV infection might also cause severe symptoms, including Guillain-Barré syndrome and congenital microcephaly (Pierson and Diamond, 2018). Currently, there is no approved specific treatment nor vaccine (Diamond et al., 2019; Bernatchez et al., 2020).

The genome of ZIKV is a positive-strand single-stranded RNA of 11 kb in length (Weaver et al., 2016; Pierson and Diamond, 2018). Once the viral genome enters cell cytoplasm, it directly encodes a polyprotein, which is processed by viral and host proteases into three structural proteins (capsid, premembrane, and envelope) and seven nonstructural proteins (NS1, NS2A, NS2B, NS3, NS4A, NS4B, and NS5). Then, nonstructural proteins such as NS4A induces ER membrane rearrangement to form viral replication complex (RC) in the perinuclear region, where viral RNA synthesis, translation, and packaging take place (Weaver et al., 2016; Pierson and Diamond, 2018).

During its replication process, ZIKV has an intensive interplay with host cells. Recently, systemic screens indicated that hundreds of candidate host proteins (Marceau et al., 2016; Savidis et al., 2016; Zhang et al., 2016; Scaturro et al., 2018) are recruited by ZIKV during its replication. Role of many proteins in ZIKV replication have been elucidated, such as α2,3-linked sialic acid which facilitates virus internalization (Tan et al., 2019), heat shock protein 70 (Pujhari et al., 2019), endoplasmic reticulum (ER) membrane protein complex (Barrows et al., 2019), adenosine deaminases acting on dsRNA 1 (Zhou et al., 2019), stearoyl-CoA desaturase-1 (Hishiki et al., 2019), and fibroblast growth factor 2 (Limonta et al., 2019). On the other hand, host cell elicits a variety of responses against ZIKV, including innate immune response, cell death, unfolded protein response, and stress granule formation, during which a number of cellular factors particularly the IFN-stimulated genes (ISG) are involved in, such as cholesterol-25-hydroxylase (Doms et al., 2018), PARP12 (Li et al., 2018), mixed-Lineage Kinase 3 (Xu et al., 2019; Yang et al., 2019), E3 ligase TRIM56 (Yang et al., 2019), and schlafen 11 (Valdez et al., 2019).

Nonetheless, more cellular proteins involved in the ZIKV replication remain to be identified. To this end, we carried out a microarray assay to monitor the transcription profiling of human lung carcinoma epithelial cells (A549) upon ZIKV infection (Ma et al., 2020). We found that expression of 139 genes was significantly upregulated by ZIKV (*p* < 0.05, change > twofold), while only one gene expression was downregulated by more than twofold. This gene is *ANKS4B* that encodes ankyrin repeat and sterile motif domain containing 4b/harmonin-interacting, ankyrin repeat-containing protein (ANKS4B). ANKS4B protein has three ankyrin repeats and a sterile motif domain (Sato et al., 2012). Functionally, ANKS4B interacts with GRP78, a major chaperone protein in the ER unfolded protein response (UPR), and regulates the ER stress-induced apoptosis in pancreatic cells (Sato et al., 2012). So far, there is no report on the interaction between ANKS4B and virus.

Current study focused on the interaction between ANKS4B and ZIKV. We found that the mRNA levels of *ANKS4B* decreased upon ZIKV infection in cultured cells (A549 and Huh7) and in neonatal mice. The downregulation of *ANKS4B* by ZIKV was caused by reduction of two transcription factors, hepatocyte nuclear factor (HNF) 1α and HNF4α. We investigated role of ANKS4B in the replication of ZIKV through loss-of-function strategy by generating two ANKS4B knockout (KO) cells. The ANKS4B KO led to an increased viral replication, while *trans*-complementation of ANKS4B suppressed the ZIKV replication, demonstrating that ANKS4B is a restriction factor of ZIKV. Moreover, we found that the autophagic process was enhanced in the ANKS4B-KO cells. Importantly, we further showed that the antiviral role of ANKS4B relies on its downregulation on the autophagy.

## Materials and Methods

### Cell Lines

Human lung carcinoma epithelial cells (A549, ATCC CCL-185), human embryonic kidney cells (293T, ATCC CRL-3216), human hepatoma cells (Huh7), and African green monkey kidney cells (Vero, ATCC CCL-81) were maintained in Dulbecco’s modified Eagle’s medium (DMEM, Gibco) supplemented with 5% or 10% fetal bovine serum (FBS) (Gibco), penicillin, streptomycin, and HEPES (Invitrogen) at 37°C in the presence of CO_2_.

### Virus, Virus Infection, and Titration

The ZIKV (H/PF/2013 strain) was provided by Guangzhou Centers for Disease Control. Virus was propagated in Vero cells. Virus stocks were titered and stored at −80°C. Cells were infected with ZIKV at a multiplicity of infection (MOI) of 3. Cells were harvested at indicated time points for western blot or real-time PCR detection. In single-step virus growth assay, the supernatants were harvested at 24 h post infection (p.i.) for virus titration as described previously (Wang et al., 2019). In multiple-step virus growth assay, cells were infected with ZIKV at an MOI of 0.01. The supernatants were harvested at 12, 24, 48, and 72 h p.i. for plaque assay.

### mRNA Microarray Data

mRNA microarray data have been deposited to the NCBI GEO database. The accession number is GSE124094.

### Quantitative Real-Time PCR (qRT-PCR)

Total cellular RNAs were extracted using TRIzol reagent (Invitrogen) and reverse transcribed using HiScript II Q RT SuperMix (Vazyme) according to the manufacturer’s protocol. The qRT-PCR analysis was performed using SYBR Premix ExTaq (TaKaRa) on a CFX96 Real-Time System (Bio-Rad). Data analysis for differences in gene expression by qRT-PCR was done by using ΔΔCT values as described previously (Wang et al., 2019). The sequences of primers used in this study were listed in Table 1.

**TABLE 1:** Sequences of primers used in qRT-PCR (“m”:mouse; “h”:human).

Gene name	Sequence (5′–3′	Product size
ZIKV NS1	5F, GTCAGAGCAGCAAAGACAA	211 bp
	3R, CATCTGCTGGAAGGTGGACA	
h–*ANKS4B*	5F, TTCTGCTCCTGGCACATTCGG	107 bp
	3R, TGCCTTCCTTCCCCACCTGTT	
h–*HNF1α*	5F,CCCCACTTGAAACGGTTC	122 bp
	3R,CTGTCCCAACACCTCAACAA	
h–*HNF4α*	5F, GACAAAGACAAGAGGAACCAGT	191 bp
	3R, TCATAGCTTGACCTTCGAGTG	
h–β–*actin*	5F, GCTCCTCCTGAGCGCAAG	75 bp
	3R, CATCTGCTGGAAGGTGGACA	
m–*ANKS4B*	5F,CCAATGGCCACACTCATTGC	196 bp
	3R,GCCTGTTCCTTCAGTCTGGT	
m–*HNF1α*	5F,CTGACCGAGTTGCCTAATGG	168 bp
	3R,TGGGTCCTCCTGAAGAAGTG	
m–*HNF4α*	5F,TGGACAGCTTCCTTCTTC	193 bp
	3R,CCACCGGCAAACACTACGGA	
m–β–*actin*	5F,ACACTGTGCCCATCTACGAG	154 bp
	3R,ATGTCACGCACGATTTCCC	

### Animal Models and Experiments

Pregnant Kunming mice were purchased and maintained under specific pathogen–free conditions at the animal facility of Sun Yat–sen University. Neonatal Kunming mice were breast–fed by their own mothers and divided into different groups. 2–day–old neonatal Kunming mice were intracerebroventricularly (i.c.v.) injected with 20 μl ZIKV(4 × 10^4^ PFUs) or PBS, and monitored over 14 days. All animals were observed daily until development of symptoms and cage-bred with the mouse mothers during the experiment. At 10 d p.i., mice tissues including brain, liver, lung, and kidney were collected and homogenized for total RNA extraction by TRIzol reagent (Invitrogen). All experiments were performed in accordance with the National Institutes of Health Guide for the Care and Use of Laboratory Animals, and the study was approved by the animal Ethics Committee of Zhongshan School of Medicine, Sun Yat-sen University (ethics reference number: 2020-000211). All experiments were operated in BSL2 lab.

### Western Blot

Western blot was performed as previously described (Wang et al., 2019). Briefly, whole cell extracts were prepared using lysis buffer (50 mM Tris–HCl, 0.5% (vol/vol) NP-40, 1% Triton-100, 150 mM NaCl, 1 mM EDTA, 1 mM PMSF, 1% protease inhibitor cocktails, 1 mM Na_3_VO_4_, and 1 mM NaF, pH 7.4). Proteins were separated on 10% SDS-PAGE and transferred onto nitrocellulose membranes or PVDF membranes. The membranes were incubated in 0.1% PBST with 5% BSA, followed by PBS washing and primary antibody incubation at 4°C overnight. The primary antibodies included: anti-ZIKV envelope (E) (GeneTex, GTX 133314), anti-p62 (Santa Cruz, sc-28359), anti-GRP78 (Proteintech, 11587-1-AP), anti-Calnexin (Proteintech, 66903-1-lg), anti-FLAG (MBL, PM020), and anti-anti-glyceraldehyde-3-phosphate dehydrogenase (GAPDH) (Proteintech, 10494-1-AP). Detection was performed with IRDye 800 CW-conjugated anti-rabbit IgG and IRDye 680 CW-conjugated anti-mouse IgG secondary antibody (LI-COR) according to the manufacturer’s protocols. Immunoreactive bands were visualized using an Odyssey infrared imaging system (LI-COR) as described previously (Doms et al., 2018).The western blot bands were quantified by Quantity One (Bio-Rad).

### Plasmid Construction

The sequences of oligonucleotide used for generation of sgRNAs were listed in Table 2. A pair of forward and reverse oligonucleotides were annealed and then inserted into plasmid vectors LentiCRISPR v2 (Addgene #52961) between *Bsm*B I restriction sites. The resulting plasmids were designated as pLenti-sgANKS4B-1 (targeting the ANKS4B gene).

**TABLE 2:** Sequences of primers used in PCR.

Gene name	Sequence (5′–3′
*ANKS4B-FLAG*	5F, ATGTACCCATACGATGTTCCAGATTACGCT
	3R, GAATTCTCACTTATCGTCGTCATCCTTGT
	3R, AATCACCACCACCCAGGCTGGTGTCGACCA
sg-ANKS4B	5F, CACCGGCCTCACATAGTTGTCCATC
	3R, AAACGATGGACAACTATGTGAGGCC
ANKS4B mutation	5F, AAAGAGGCTACTAAACGAGATCTAAATC
	3R, GATTTAGATCTCGTTTAGTAGCCTCTTT

The *ANKS4B* gene was amplified by PCR using A549 cDNA as template. The PCR primer sequences were listed in Table 2. PCR product was inserted into pSG5 vector or a lentiviral vector CSII–EF–MCS–IRES2–Venus and sequenced as described previously (Gao et al., 2019). The resulting plasmids were designated as pSG5–ANKS4B–FLAG and pCSII–EF–MCS–IRES2–Venus–ANKS4B. pCSII–EF–MCS–IRES2–Venus–ANKS4B was mutated at synonymous sites in sgRNA–targeting sequence present in the human *ANKS4B* ORF in order to resist the gene editing. The mutations were verified by DNA sequencing.

### Generation of ANKS4B KO Cell Clones and ANKS4B–RES Cells

CRISPR/Cas9 system was utilized to generate ANKS4B–KO cell clones as described previously (Gao et al., 2019; Wang et al., 2019). Briefly, 293T cells were transfected with pLenti–sgANKS4B–1and packaging plasmids (psPAX2 and pMD2.G) using FuGENE HD Transfection Reagent (Promega). At 48 h post transfection, supernatants were collected and passed through a 0.45 μm filter. The lentivirus supernatants were transduced into A549 or Huh7 cells for 24 h. Then cells were transferred to 10–cm dishes and selected by 1 μg/ml puromycin. Puromycin–resistant clones were sorted and confirmed by genomic DNA sequencing and qRT–PCR. Regions surrounding sgRNA–targeting sequences were amplified by PCR using genomic DNA as template. PCR products were cloned into pMD–18T (Takara) for sequencing as described previously (Wang et al., 2019). qRT–PCR was performed using the primers located in the sgRNA–targeting region.

To generate ANKS4B–complemented cells, 293 T cells were co–transfected with CSII–EF–MCS–IRES2–Venus–ANKS4B, pSPAX2, and pMD2.G using FuGENE HD Reagent (Promega) for lentivirus packaging. After 2 days, supernatants were collected. The ANKS4B–KO cells were transduced with lentivirus carrying *ANKS4B* gene. Venus positive cells were sorted by flow cytometry (CytoFLEX). The purity of ANKS4B–RES cells was estimated to be more than 80%.

### Co–IP Assay

To detect the endogenous ANKS4B protein, control and ANKS4B–KO cells were collected in 1 ml ice–cold RIPA lysis buffer with protease inhibitors (Sigma) and phosphatase inhibitor (NaF and Na_3_VO_4_). Co–IP assay was performed using monoclonal anti–ANKS4B antibody (Invitrogen).

To detect the interaction between ANKS4B and GRP78, A549 and 293T cells were transfected with plasmids expressing ANKS4B–FLAG fusion protein by Lipofectamine 2000 reagent (Invitrogen). At 24 h post transfection, cells were resuspended in RIPA lysis buffer and applied for co–IP assay using monoclonal anti–FLAG agarose (Sigma).

Cells lysates were spun for 30 min at 4°C, followed by incubation with 20 μl of monoclonal anti-FLAG agarose (Sigma) at 4°C overnight. Beads were washed with NET-RIPA wash buffer (50 mM Tris–HCl, 0.5% NP-40, 1 mM EDTA, 150 mM NaCl), and proteins were eluted in loading buffer.

### Viral Entry Assay

In the viral attachment assay, cells were incubated with ZIKV at MOI 3 at 4°C for 1 h. The supernatants of cells were discarded, followed by washing with PBS buffer. In the endocytosis assay, cells were incubated with viral particles at MOI of 3 at 37°C for 1 h to allow viral attachment and internalization. The supernatants of cells were then discarded, followed by washing with PBS for three times. Total RNAs were extracted with TRIzol reagent (Invitrogen) and viral RNA levels were detected by qRT-PCR.

### Autophagy Inhibitor Treatment

Two inhibitors of autophagy, chloroquine (CQ) and 3-methyladenine (3-MA), were dissolved in DMSO at a stock concentration of 100 mM (CQ) or 200 mM (3-MA). The CQ (50 μM) and 3-MA (5 mM) working solution was freshly prepared before use. Growth media and 0.1% DMSO were used as parental and vehicle controls respectively. Cells were infected with ZIKV at MOI 3. At 1 h p.i., cells were added with CQ throughout the following infection. At 24 h p.i., cell lysates were prepared for western blot and supernatants were collected for plaque assay.

### Statistical Analysis

All the data were shown as means ± standard deviations (S.D.) from at least three independent experiments. The statistical analysis was performed with an unpaired, two-tailed Student’s *t*-test.

## Results

### Expression Level of ANKS4B Is Decreased Upon ZIKV Infection

**FIGURE 1:**
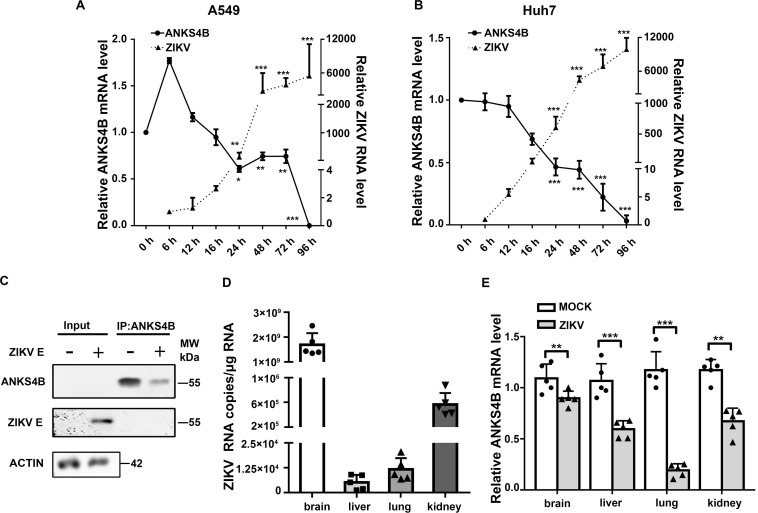
Expression of ANKS4B was reduced by ZIKV in vitro and *in vivo*. **(A,B)** Quantitative RT-PCR analysis (qRT-PCR). A549 **(A)** or Huh7 cells **(B)** were infected with ZIKV (MOI 3), and collected at indicated time points for qRT-PCR. *GAPDH* mRNA levels were set an internal control. Relative RNA levels were compared to the RNA level measured at 0 h p.i. **(C)** co-IP assay. Huh7 cells were infected with ZIKV (MOI 3), and collected at 24 h p.i. for co-IP assay using anti-ANKS4B antibody. Protein complex was separated by SDS-PAGE and detected by western blot with antibodies against ANKS4B, ZIKV E, and β-actin. **(D,E)** qRT-PCR to measure the levels of ZIKV and ANKS4B in mice tissues (*n* = 5 per group). PBS or ZIKV was intracerebroventricularly injected into Kunming neonatal mice on day 2 after birth. Tissues including brain, liver, lung, and kidney were collected at 10 d p.i. for total RNA extraction. Viral RNA copies were determined by qRT-PCR. Expression levels of *ANKS4B* were measured by qRT-PCR, and normalized to *β-actin* levels. Relative RNA levels were calculated over the RNA level measured at 0 h p.i. All the data were shown as means ± S.D. (error bars) from at least three independent experiments. NS, not significant; *p < 0.05; **p < 0.01; ***p < 0.001; unpaired, two-tailed Student’s *t*-test.

Our previous work revealed that in A549 cells, only one gene (*ANKS4B*) expression was decreased by more than twofold upon ZIKV infection (Ma et al., 2020). Present study first validated the microarray data by qRT-PCR measuring temporal expression of *ANKS4B* in the ZIKV-infected A549 cells. Cells were infected with mock or ZIKV at MOI 3, and harvested at indicated time points for qRT-PCR. As shown in Figure 1A, the ZIKV RNA levels were increased with time extended, indicating the viral infection was successful. The expression of *ANKS4B* was slightly increased at 6 h p.i., but then significantly decreased at all later time points (Figure 1A). At 24, 48, and 72 h p.i., the mRNA levels of *ANKS4B* in the ZIKV-infected cells were about half of those in the mock-infected cells, consistent with our microarray data (Ma et al., 2020).To be noted, the mRNA level of *ANKS4B* was extremely low at 72 h p.i., probably due to the cytopathic effect of viral infection. As *ANKS4B* has been reported to be abundantly expressed in liver, kidney, small intestine, and colon (Johnston et al., 2004), we examined whether ZIKV infection had an impact on ANKS4B expression in a hepatoma cell line, Huh7. The mRNA levels of *ANKS4B* in the ZIKV-infected Huh7 cells were also gradually decreased over time (Figure 1B). In addition, we conducted the co-IP assay to enrich endogenous ANKS4B protein in Huh7 cells at 24 h p.i. The data confirmed that the protein level of ANKS4B in the ZIKV-infected cells was significantly reduced (Figure 1C). These observations suggested the downregulation of ANKS4B mRNA levels by ZIKV infection is not cell-specific.

Furthermore, we examined whether the *ANKS4B* expression was modulated by ZIKV infection in a neonatal mice model. The neonatal mice were infected with ZIKV, and the tissues were collected at 10 d p.i. for qRT-PCR. The data showed that the viral RNA copies in brains were dramatically higher than those in livers, lungs, and kidneys (Figure 1D). Interestingly, the *ANKS4B* levels were significantly reduced in all ZIKV-infected tissues (Figure 1E), indicating that ZIKV infection also led to *in vivo* downregulation of ANKS4B.

### ZIKV Infection Downregulates Levels of ANKS4B Through Decreasing the Levels of HNF1a and HNF4a

**FIGURE 2:**
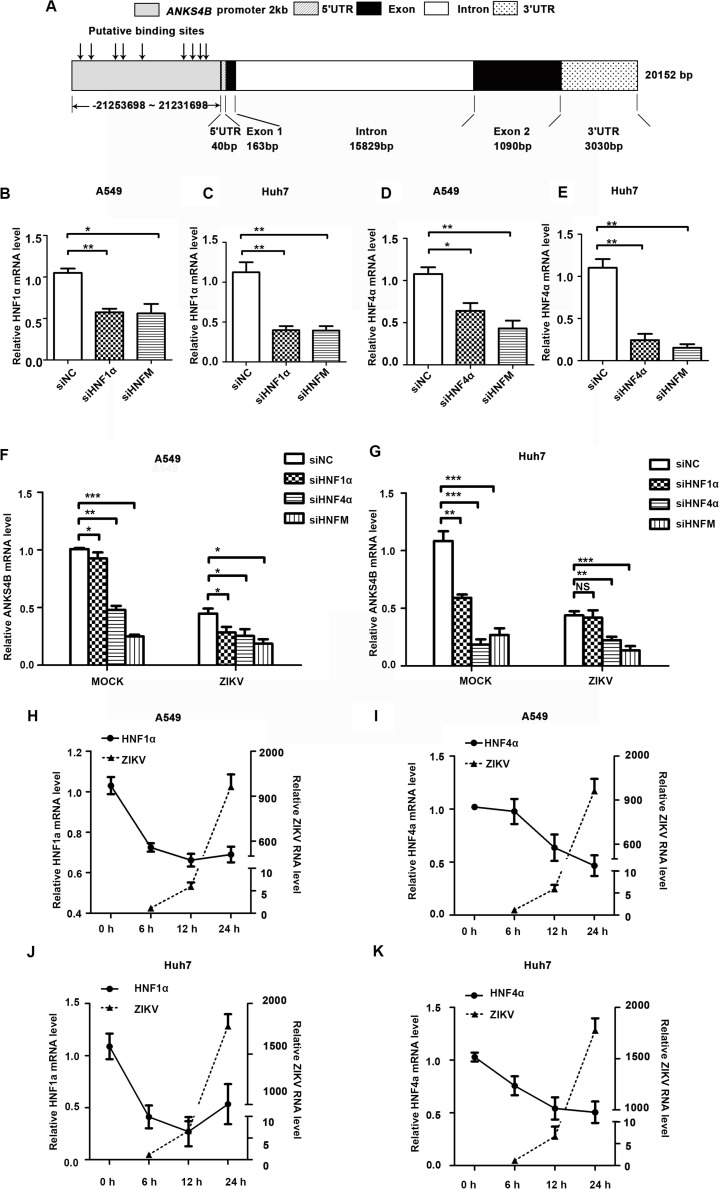
Reduction of ANKS4B by ZIKV infection was dependent of HNF1α and HNF4α. **(A)** Putative HNF1α and HNF4α binding sites in the promoter region of *ANKS4B*. **(B–G)** Effect of *HNF1α* and *HNF4α* knockdown on *ANKS4B* level. A549 cells and Huh7 cells were transfected with siNC, siHNF1α, siHNF4α, or siHNFm (mixture of siHNF1α and siHNF4α). At 48 h post-transfection, cells were harvested for qRT-PCR to detect the levels of *HNF1α* **(B,C)**, *HNF4α* **(D,E)**, and *ANKS4B* **(F,G)**. **(H–K)** qRT-PCR to detect *HNF1α* and *HNF4α* levels in A549 **(H,I)** or Huh7 **(J,K)** cells. Cells were infected with ZIKV (MOI 3), and collected at indicated time points for qRT-PCR. *GAPDH* level was set as an internal control. Relative RNA levels were calculated over the RNA level measured at 0 h p.i. All the data were shown as means ± S.D. (error bars) from at least three independent experiments. NS, not significant; *p < 0.05; **p < 0.01; ***p < 0.001; unpaired, two-tailed Student’s *t*-test.

To explore how the expression of ANKS4B was downregulated by ZIKV infection, we carried out bioinformatics analysis to predict potential binding sites of transcription factors in its promoter region using an online software. As shown in Figure 2A, nine putative binding sites of HNF1α and HNF4α were predicted, consistent with a previous report (Sato et al., 2012). To examine whether HNF1α and HNF4α regulates the transcription of *ANKS4B* in A549 and Huh7 cells, we first compared the *ANKS4B* levels in the control and HNF1α/HNF4α knockdown cells. Cells were transfected with siNC, siHNF1α, siHNF4α, or their combination, followed by ZIKV infection. Cells were collected at 24 h p.i. for qRT-PCR. Transfection of siHNF1α alone led to approximately 50% knockdown effect on HNF1α level in A549 (Figure 2B) and Huh7 (Figure 2C) cells, and siHNF4α caused 36.1 and 75.6% reduction of HNF4α levels in A549 (Figure 2D) and Huh7 (Figure 2E) cells. Transfection of the siRNA mixtures showed synergistic effect on both genes. In general, knockdown efficiency of siRNAs was higher in Huh7 cells than in A549 cells, probably because the endogenous levels of HNFs in liver Huh7 cells were much higher (Cereghini, 1996).The levels of *ANKS4B* in mock- and ZIKV-infected cells were significantly lower in *HNF4α* knockdown cells (Figures 2F,G). Interestingly, transfection of the siHNF1α and siHNF4α 4 mixture led to even more reduction of *ANKS4B* expression, suggesting that *HNF4α* regulates the transcription of *ANKS4B*, and *HNF1α* has a synergistic effect.

Next, we explored whether the ZIKV-mediated *ANKS4B* reduction is dependent of the HNFs levels. A549 cells and Huh7 cells were infected with ZIKV, and harvested at 6, 12, and 24 h for qRT-PCR. In these cells, the levels of *HNF1α* and *HNF4α* were gradually decreasing after infection (Figures 2H–K). At 24 h p.i., the levels of HNFs in A549 or Huh7 cells were reduced by 1.3–2.2 fold compared to mock-infected cells, suggesting that ZIKV infection downregulated the levels of HNFs, which further led to the decrease of *ANKS4B*.

### Replication Levels of ZIKV Are Enhanced in ANKS4B KO A549 Cells

**FIGURE 3:**
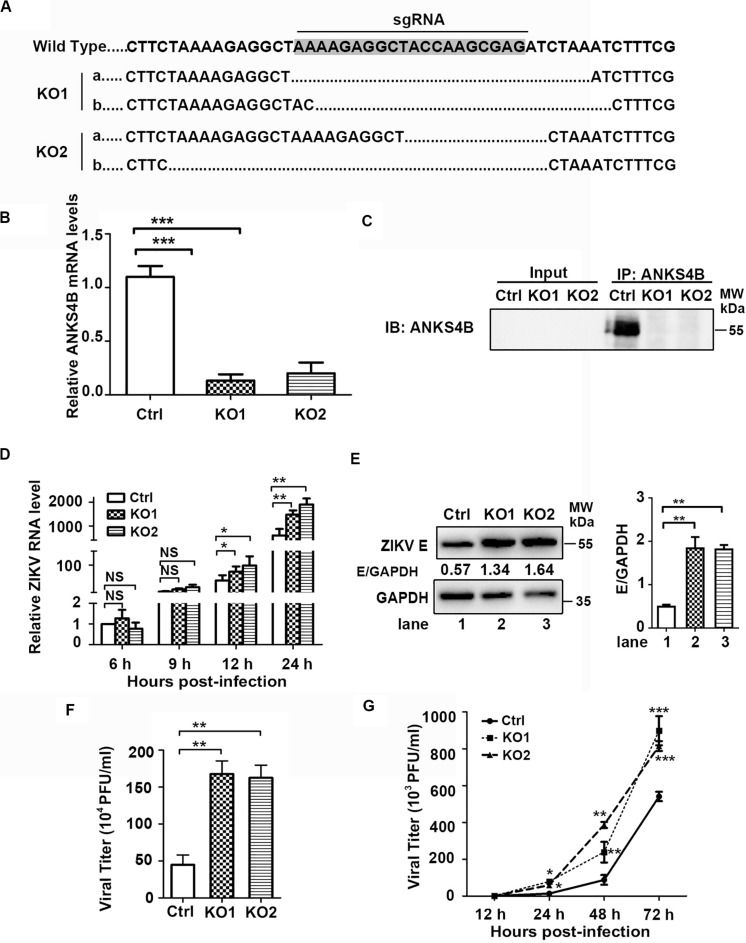
ANKS4B inhibited the replication of ZIKV in A549 cells. **(A–C)** Confirmation of *ANKS4B* knockout efficiency. The genomic DNA, total RNAs, and whole cell extracts of ANKS4B-KO1 and ANKS4B-KO2 A549 cells were extracted for DNA sequencing **(A)**, qRT-PCR assay **(B)**, and co-IP assay **(C)** using anti-ANKS4B antibody. Protein complexes were separated by SDS-PAGE and detected by western blot with indicated antibodies. **(D–F)** Replication levels of ZIKV. Control and ANKS4B-KO cells were infected with ZIKV (MOI 3) and collected at 6, 12, and 24 h p.i. The viral RNA levels were measured by qRT-PCR. *GAPDH* mRNA level was measured as an internal control **(D)**. At 24 h p.i., western blot was performed to detect levels of ZIKV E protein and GAPDH **(E)**. Supernatants were harvested at 24 h p.i. for plaque assay **(F)**. **(G)** Multiple step virus growth assay. Control and ANKS4B-KO cells were infected with ZIKV at MOI 0.01. At 24, 48, and 72 h p.i., supernatants were collected for plaque assay. All the data were shown as means ± S.D. (error bars) from at least three independent experiments. NS, not significant; *p < 0.05; **p < 0.01; ***p < 0.001; unpaired, two-tailed Student’s *t*-test.

To investigate whether ANKS4B has an impact on ZIKV replication, we utilized CRISPR/Cas9 gene editing system to generate two ANKS4B-KO cell clones, designated as ANKS4B-KO1 and ANKS4B-KO2. Disruption of ANKS4B expression was confirmed by sequencing and qRT-PCR. The sequencing data showed that the genomic DNAs in ANKS4B gene of KO cells were successfully edited (Figure 3A), and the mRNA levels of ANKS4B in both KO cells were substantially lower than the control cells (Figure 3B). As the ANKS4B protein could not be detected by direct SDS-PAGE and western blot probably due to the low abundance of ANKS4B or low reactivity of antibody, we carried out a co-IP assay to enrich ANKS4B protein, followed by western blot. ANKS4B was readily detected in the control cells, but not in two KO cells, further confirming the gene-editing was successful (Figure 3C). Then, we carried out ZIKV infection assay and compared the viral RNA levels, protein accumulation, and titers in the control and ANKS4B-KO cells. Cells were infected with mock or ZIKV at MOI 3. Total RNAs were extracted at 6, 9, 12, and 24 h p.i. The qRT-PCR data showed that at early time points (6 and 9 h p.i.), the viral RNA levels were similar in all tested samples, while at 12 and 24 h p.i., the viral RNA levels in ANKS4B-KO1 and ANKS4B-KO2 cells were increased by around 2.17 and 3.17 fold respectively (Figure 3D). Next, western blot and plaque assay were performed at 24 h p.i. Higher E protein levels in both ANKS4B-KO cells were evidently observed (Figure 3E). Similarly, viral yields in the ANKS4B-KO cells were enhanced by more than threefold (*p* < 0.01, Figure 3F).

Furthermore, we conducted a multiple-step virus growth assay to examine whether ANKS4B affects the transmission of ZIKV. The control and ANKS4B-KO cells were infected with ZIKV at an MOI of 0.01, and supernatants were collected at 24, 48, and 72 h p.i. for plaque assay. The data showed that at 24, 48, and 72 h p.i., the viral titers in the ANKS4B-KO cells were all markedly increased (Figure 3G). These data indicated that ANKS4B restricts the replication and transmission of ZIKV in A549 cells.

### ANKS4B Plays an Antiviral Role in Huh7 Cells

**FIGURE 4:**
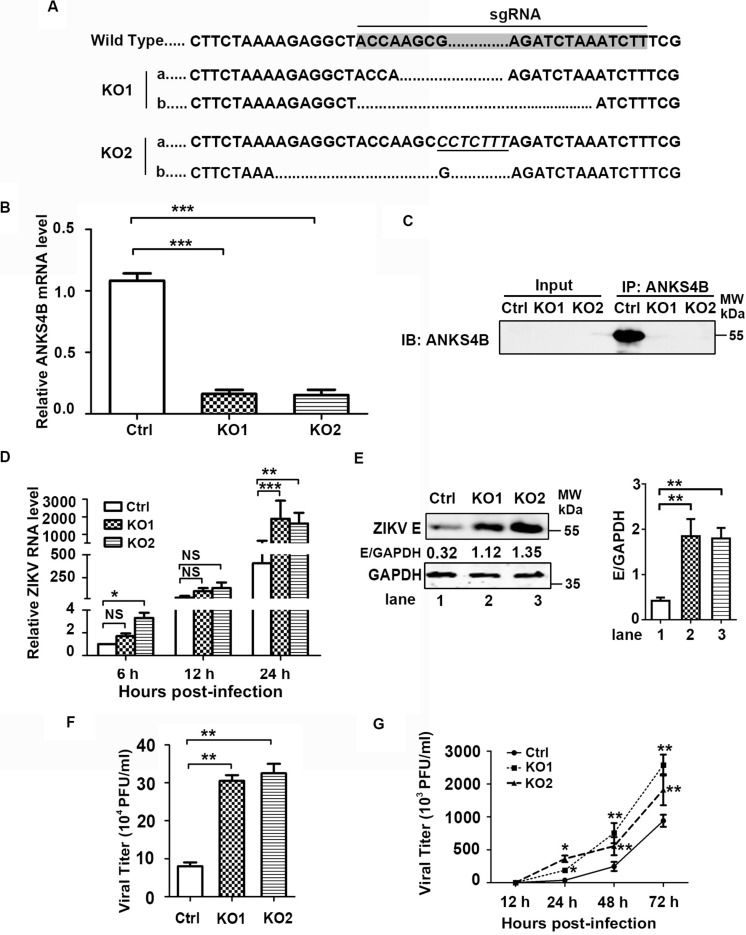
ANKS4B inhibited the replication of ZIKV in Huh7 cells. **(A–C)** Confirmation of ANKS4B knockout efficiency. The genomic DNAs, total RNAs, and whole cell extracts of ANKS4B-KO1 and ANKS4B-KO2 A549 cells were extracted for DNA sequencing **(A)**, qRT-PCR assay **(B)**, and co-IP assay **(C)**. Italics indicated inserted bases. Co-IP assay was performed using anti-ANKS4B antibody. Protein complexes were separated by SDS-PAGE and detected by western blot with indicated antibodies. **(D–F)** Replication levels of ZIKV. Control and ANKS4B-KO cells were infected with ZIKV (MOI 3) and collected at 6, 12, and 24 h p.i. The viral RNA levels were measured by qRT-PCR. *GAPDH* mRNA level was measured as an internal control **(D)**. Western blot was performed to detect ZIKV E protein level and GAPDH **(E)**. The supernatants were harvested at 24 h p.i. for plaque assay **(F)**. **(G)** Multiple step virus growth assay. Control and ANKS4B-KO cells were infected with ZIKV at MOI 0.01. At 24, 48, and 72 h p.i., supernatants were collected for plaque assay. All the data were shown as means ± S.D. (error bars) from at least three independent experiments. NS, not significant; *p < 0.05; **p < 0.01; ***p < 0.001; unpaired, two-tailed Student’s *t*-test.

As ANKS4B is abundantly expressed in hepatoma cells, we then investigated whether ANKS4B confers an antiviral activity in Huh7 cells. Two ANKS4B KO cell clones of Huh7 (ANKS4B-KO1 and ANKS4B-KO2) were generated by CRISPR/Cas9 gene editing system. Sequencing and qRT-PCR data confirmed the genomic ablation of ANKS4B gene in these two cell clones (Figures 4A,B). The co-IP and western blot data verified that ANKS4B protein was expressed in the control cells, but not in two ANKS4B-KO cells (Figure 4C). Then, we infected the control cells and ANKS4B-KO cells at MOI 3. Total RNAs were prepared at 6, 12, and 24 h p.i. for qRT-PCR. At early time points, the viral RNA levels were comparable in the control and ANKS4B-KO cells. In contrast, more than threefold higher viral RNA levels in two ANKS4B-KO cells were observed at 24 h p.i. (Figure 4D). Next, cells and supernatants were harvested at 24 h p.i. for western blot and plaque assay. In the absence of ANKS4B, higher E protein accumulation were detected (Figure 4E), and around threefold higher viral particles were produced, in agreement with the above observations in A549 cells (Figure 4F). In the multiple-step virus growth assay, ANKS4B KO led to significantly higher viral titers in both cell clones at 1, 2, and 3 days p.i. (Figure 4G). These results indicated that ANKS4B also limits the ZIKV replication and transmission in hepatocytes.

### *Trans*-Complementation of ANKS4B Downregulates the ZIKV Replication

**FIGURE 5:**
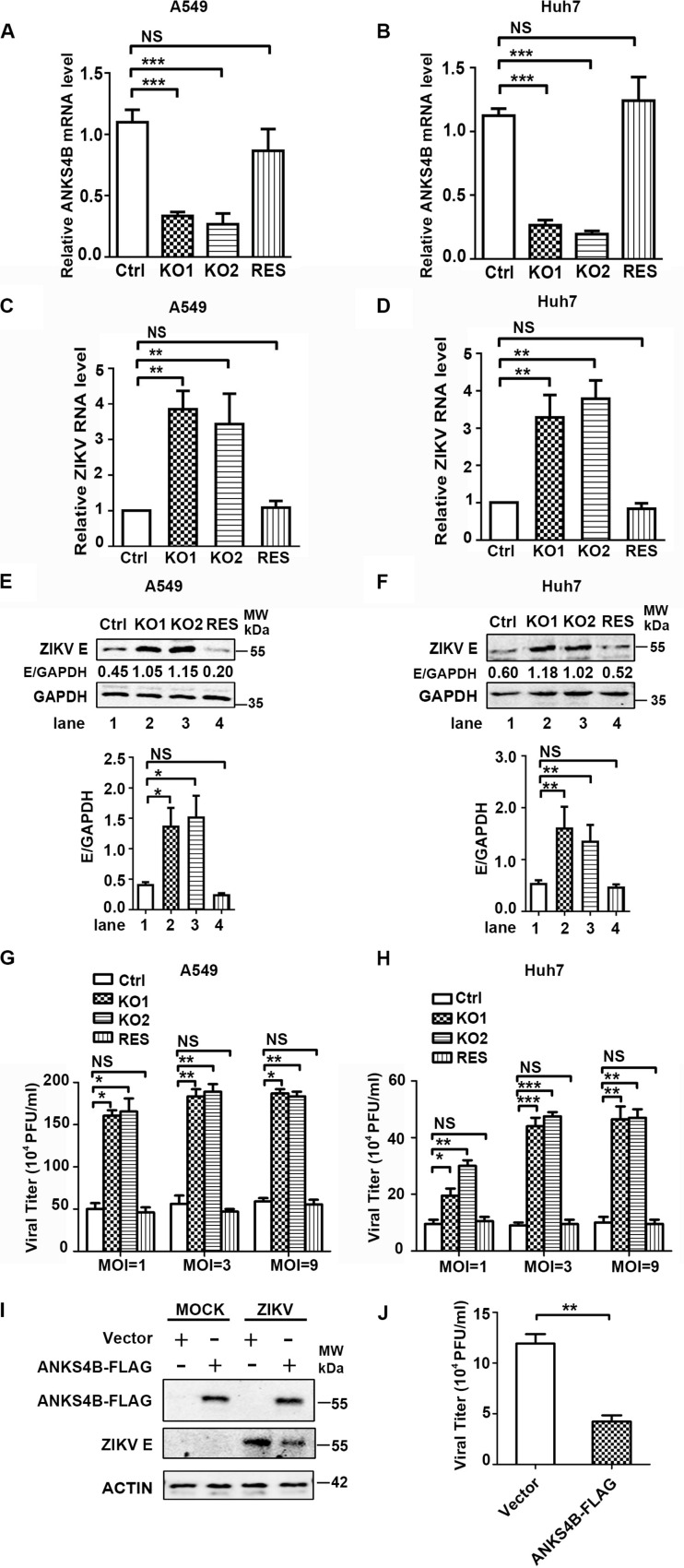
Complementation of *ANKS4B* rescued the viral replication suppressed by ANKS4B KO. **(A,B)** The ANKS4B complementation in ANKS4B-KO A549 **(A)** and Huh7 cells **(B)** were confirmed by qRT-PCR. **(C–H)** viral replication levels. Control, ANKS4B-KO1, ANKS4B-KO2, and ANKS4B-RES cells were infected with ZIKV (MOI 3). At 24 h p.i., viral RNA level in A549 **(C)** or Huh7 **(D)** cells was measured by qRT-PCR and normalized to GAPDH. Whole cell extracts were subjected to western blot to detect ZIKV E protein levels **(E,F)**. GAPDH was probed as an internal control. Cells were infected by ZIKV at different MOIs (1, 3, and 9). At 24 h p.i., supernatants were collected for plaque assay **(G,H)**. **(I,J)** Effect of ANKS4B overexpression on viral replication. Huh7 cells were transfected with vector or plasmid expressing ANKS4B-FLAG protein, followed by ZIKV infection at 24 post transfection. Cells and supernatants were harvested at 24 h p.i. for western blot or plaque assay. GAPDH was probed as an internal control. All the data were shown as means ± S.D. (error bars) from at least three independent experiments. NS, not significant; *p < 0.05; **p < 0.01; ***p < 0.001; unpaired, two-tailed Student’s *t*-test.

To further confirm the antiviral role of ANKS4B in ZIKV replication, we introduced *ANKS4B-FLAG* fusion gene into the ANKS4B-KO cells (A549 and Huh7) through lentivirus-mediated transduction. The ANKS4B-restored cells (ANKS4B-RES) were sorted by flow cytometry. Complementation of ANKS4B was validated by qPCR (Figures 5A,B). Then, the viral replication levels including RNA, protein, and titer in control, ANKS4B-KO, and ANKS4B-RES cells were measured by qRT-PCR, western blot, and plaque assay respectively. Cells were infected with ZIKV at MOI 3, and harvested at 24 h p.i. In both ANKS4B-KO A549 and Huh7 cells, three- to fourfold enhancement of viral RNA levels were observed (Figures 5C,D). As expected, the enhancement of viral RNA levels in the ANKS4B-KO cells was reversed by restoration of ANKS4B. Similar levels of viral replication were detected in the control and ANKS4B-RES cells (Figures 5C,D). Moreover, the western blot analysis revealed that in both A549 and Huh7 cells, complementation of ANKS4B successfully downregulated viral E protein levels compared to the ANKS4B-KO cells (Figures 5E,F). Consistently, restoration of ANKS4B reduced the viral yields by three- to fourfolds compared to the ANKS4B-KO cells at all tested MOIs, including MOI 1, MOI 3, and MOI 9 (Figures 5G,H). Furthermore, we examined whether the replication levels of ZIKV could be regulated by transient overexpression of ANKS4B. Huh7 cells were transfected with control vector or ANKS4B-FLAG expressing plasmid, followed by ZIKV infection. Our data revealed that at 24 h p.i., the viral E protein levels and viral yields in the ANKS4B-FLAG expressing cells were significantly downregulated (Figures 5I,J), providing another piece of evidence that ANKS4B plays an antiviral role.

### ANKS4B Does Not Affect the Viral Entry Step

**FIGURE 6:**
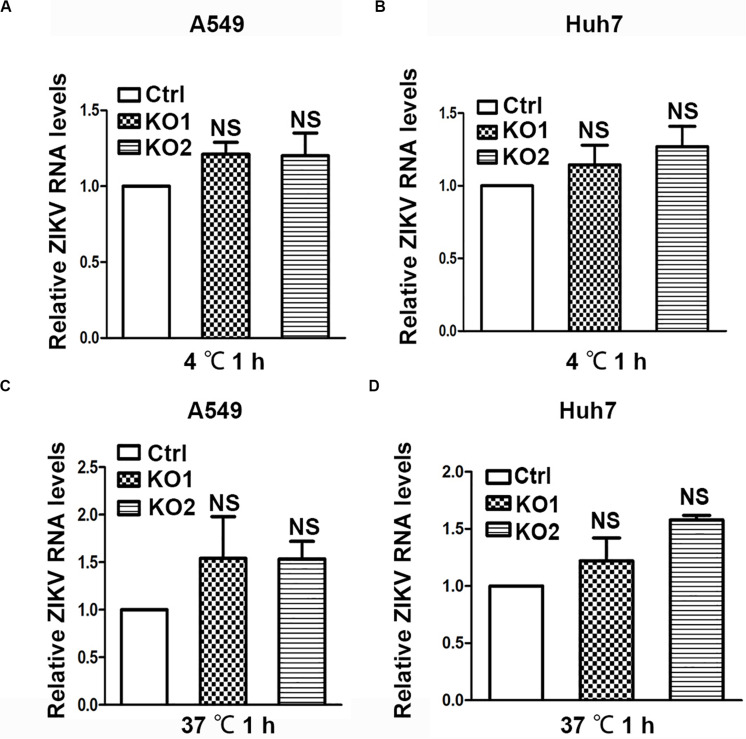
ANKS4B did not block the viral attachment or endocytosis step. **(A,B)** Effect of ANKS4B on viral attachment step. A549 and Huh7 cells were infected with ZIKV at MOI 3 at 4°C for 1 h. Cells were then washed with PBS and harvested for RNA extraction. **(C,D)** Effect of ANKS4B on ZIKV endocytosis process. A549 and Huh7 cells were inoculated with ZIKV at MOI 3 at 37°C for 1 h, followed by RNA extraction. qRT-PCR assay was performed to detect viral RNA levels. GAPDH level was measured as an internal control. All the data were shown as means ± S.D. (error bars) from at least three independent experiments. NS, not significant. unpaired, two-tailed Student’s *t*-test.

As ANKS4B is associated with cell membrane and ER membrane (Sato et al., 2012), we examined whether it blocks attachment or endocytosis step of ZIKV. We inoculated the ZIKV virions onto control and ANKS4B-KO cells, and incubated at 4°C for 1 h to allow viral attachment, in which condition internalization of virions was blocked. Unattached virions were extensively washed away. Total cellular RNA were prepared for qRT-PCR to measure the amount of attached virions. As shown in Figure 6A (A549) and Figure 6B (Huh7), similar levels of viral RNA were observed in the control and ANKS4B-KO cells, suggesting that ANKS4B does not regulate the viral attachment. Next, cells were inoculated with ZIKV at 37°C to let virions attach and be internalized. At 1 h post inoculation, cells were extensively washed and collected for qRT-PCR to measure the amount of internalized virions. In Figures 5C,D, the viral RNA levels in control and ANKS4B-KO cells were also comparable (*p*> 0.05), suggesting that ANKS4B does not affect the viral endocytosis step.

### The Antiviral Role of ANKS4B Relies on Its Inhibitory Effect on Autophagy

**FIGURE 7:**
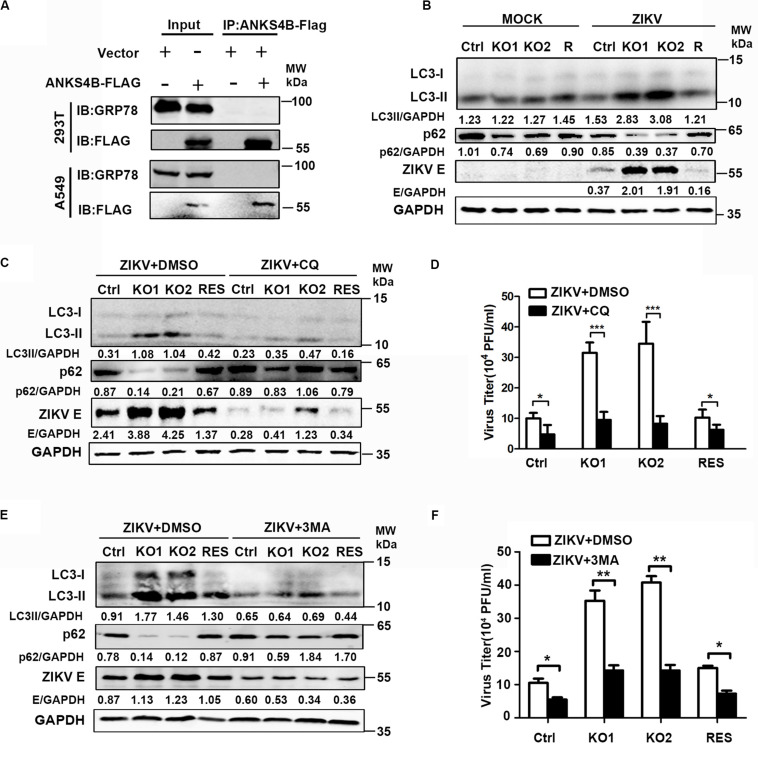
The antiviral effect of ANKS4B was exerted through inhibiting the autophagy. **(A)** Co-IP assay. A549 and 293T cells were co-transfected with plasmids expressing ANKS4B-FLAG protein and GRP78. Whole cell extracts were prepared for Co-IP assay using anti-FLAG. **(B)** Control, ANKS4B-KO1, ANKS4B-KO2, and ANKS4B-RES (R) Huh7 cells were infected with ZIKV (MOI 3). Cell lysates were prepared at 24 h p.i for western blot to detect the expression levels of ZIKV E protein, LC3B, and p62. **(C–F)** Autophagy inhibitor assay. Control, ANKS4B-KO1, ANKS4B-KO2, and ANKS4B-RES cells were treated with DMSO or chloroquine (CQ, 50 μM) or 3-methyladenine (3-MA, 5 mM) after ZIKV inoculation. At 24 h p.i., cells and supernatants were harvested for western blot **(C,E)** or plaque assay **(D,F)**. Levels of p62, ZIKV E, and GAPDH were measured. All the data were shown as means ± S.D. (error bars) from at least three independent experiments. NS, not significant. *p < 0.05; **p < 0.05; ***p < 0.001; unpaired, two-tailed Student’s *t*-test.

ANKS4B has been shown to locate near endoplasmic reticulum (ER) and interact with GRP78, a chaperon involved in ER stress and unfolded protein response (UPR) in pancreatic β-cells (Sato et al., 2012). Considering that UPR plays a role in the replication of ZIKV (Sato et al., 2012), we tested whether ANKS4B could interact with GRP78 and modulate the UPR in A549 cells by co-immunoprecipitation (co-IP) assay. 293T or A549 cells were co-transfected with plasmids expressing GRP78 and ANKS4B-FLAG fusion protein. Whole cell extracts were prepared at 24 h post transfection for co-IP assay using anti-FLAG antibody. Unfortunately, we failed to detect an interaction between ANKS4B and GRP78 in these cells after many attempts (Figure 7A), implying that the inhibitory effect of ANKS4B on ZIKV replication was independent of GRP78.

As the ER stress induced by ZIKV infection is associated with autophagy (Ojha et al., 2019), we explored whether ANKS4B has an impact on the autophagy induced by ZIKV. Cells were infected with ZIKV, and harvested at 24 h p.i. for western blot using the antibodies against microtubule-associated protein light chain 3 (LC3) and p62/SQSTM (p62). The conversion of LC3-I to LC3-II, and degradation of p62 are indicators of autophagy. As shown in Figure 7B, the protein levels of LC3-I, LC3-II, and p62 were comparable in the mock-infected cells. Upon ZIKV infection, conversion of LC3-I to LC3-II in two ANKS4B-KO cells was significantly higher than control and ANKS4B-RES cells. Consistently, the p62 levels in ANKS4B-deficient cells were notably lower than the ANKS4B-sufficient cells (Figure 7B). These data suggested that ANKS4B inhibits the autophagic process induced by ZIKV.

To examine impact of autophagy on ZIKV replication in Huh7 cells, we tested whether two inhibitors of autophagy, CQ and 3-MA affected the ZIKV replication (Abernathy et al., 2019). To avoid the interference of CQ on endosomal viral RNA release (Zhang et al., 2019), we treated cells with CQ at 1 h p.i. to allow viral genome release. Cells were incubated in the presence of DMSO or CQ throughout the infection, and harvested for western blot at 24 h p.i., DMSO treatment alone had no effect on the autophagy extents. Treatment of CQ led to lower LC3-I/LC3-II conversion and less p62 degradation in all tested cells, confirming it was effective in blocking the autophagy (Figure 7C). Consequently, the E protein levels on all CQ-treated were lower than in the DMSO-treated cells (Figure 7C), indicating the autophagy plays a proviral role in this infection model. In the presence of DMSO, higher autophagy extent and more E proteins were observed in ANKS4B KO cells as expected (Figure 7C). In contrast, in the presence of CQ, the LC3-I/LC3-II conversion, p62 degradation, and E protein levels were comparable among all tested cells, suggesting that the antiviral effect of ANKS4B relied on the occurrence of autophagy. Consistently, the treatment of CQ led to similar viral yields in control and ANKS4B-KO cells (Figure 7D).

Furthermore, we utilized another widely-used inhibitor of autophagy, 3-MA, and performed in a same procedure as CQ except the concentration of 3-MA was 5 mM. The treatment of 3-MA also significantly downregulated the LC3-I/LC3-II conversion and p62 degradation, indicating it effectively inhibited the autophagy process induced by ZIKV (Figure 7E). In the ANKS4B-KO cells, the 3-MA treatment resulted in lower E protein levels (Figure 7E) and viral titers (Figure 7F) compared to the DMSO treatment, suggesting that autophagy mediates the ANKS4B antiviral-effect. Altogether, these data suggested that ANKS4B suppresses the autophagy, and hence restricts the ZIKV replication.

## Discussion

Hundreds of cellular proteins are involved in ZIKV life cycle. Some proteins are employed by virus, while other proteins act as a defense against viral replication. In current study, we uncovered an interaction between ZIKV and ANKS4B, which functions are largely unknown.

First, we demonstrated that the expression of ANKS4B was downregulated by ZIKV infection. The microarray data revealed that in the ZIKV-infected A549 cells, only the mRNA level of ANKS4B was downregulated by more than twofold (Ma et al., 2020). We further revealed that downregulation of ANKS4B by ZIKV was in a time-dependent manner, in both lung epithelial cells (A549) expressing low-abundance of ANKS4B and liver cells (Huh7) expressing high-abundance of ANKS4B. In *in vivo* mice model, the ANKS4B expression was also downregulated upon ZIKV infection, implying that the modulation of ANKS4B by ZIKV is not cell specific.

Consistent with previous report that the ANKS4B was transcriptionally regulated by HNF1α and HNF4α in pancreatic cells (Sato et al., 2012), *ANKS4B* levels in A549 and Huh7 cells were also correlated with *HNF1α* and *HNF4α*. Knockdown of both HNFs simultaneously resulted in a more significant reduction of *ANKS4B* than knockdown of single HNF, further supporting previous notion that they cooperatively promote the target gene (Ozeki et al., 2001). In our qRT-PCR assay, the levels of *HNF1α* and *HNF4α* in A549 cells were downregulated by 1.75–2.32 fold, while in the microarray data, their levels were not significantly downregulated. These discrepancies might be because the endogenous levels of *HNF1α* and *HNF4α* in A549 cells are extremely low so that small change could not be captured by array. Moreover, the downregulation of HNFs was consistently observed in the Huh7 cells expressing abundant HNFs. Therefore, we proposed that the decrease of *ANKS4B* in the ZIKV infected cells was due to reduced levels of its transcriptional factors, *HNF1α* and *HNF4α*.

Significantly, ANKS4B plays an antiviral role in the ZIKV replication. Through loss-of-function strategy, the KO of ANKS4B in both A549 and Huh7 cells enhanced the viral RNA levels, protein, and titers by more than threefold. In addition, the *trans*-complementation of ANKS4B completely restores their phenotypes: the ZIKV replication levels were reduced to a same level as control cells. As no literature reported that ANKS4B is upregulated by type I or type III IFNs, in combination with our observations that its expression was decreased while expression of IFNs and traditional ISGs such as Mx1 and PKR were upregulated (Ma et al., 2020), we concluded that the antiviral action of ANKS4B is independent of IFN system.

We excluded a role of ANKS4B in blocking viral entry. In ANKS4B-sufficient and -deficient cells, similar amounts of virions attached and were internalized during 1 h incubation at 4°C or 37°C (Figure 6); additionally, similar RNA levels at early time points (6 and 9 h p.i., Figures 3D, 4D) were achieved, providing another piece of evidence that same amounts of genomic RNA enter these cells. So far, we could not determine at which stage that ANKS4B acts: RNA replication, protein translation, packaging, or egress. Future study utilizing a ZIKV replicon system will promote our understanding.

Unexpectedly, no interaction between ANKS4B and GRP78 was detected in A549 cells as found in pancreatic β-cells (Sato et al., 2012). Although we have repeated these assays for several times with different conditions in both 293T and A549 cells, the results were consistently negative. To be pointed out, we did not observe different cytopathic effects and apoptosis in ANKS4B-sufficient and -deficient cells infected by ZIKV, although ANKS4B regulates the cell sensitivity to ER stress and apoptosis via interacting with GRP78 in pancreatic cells (Sato et al., 2012). Obviously, functions of ANKS4B in UPR vary in response to different stimuli in different cells. ANKS4B does not play a key role in A549 cells as in pancreatic β-cells, implying alternative regulation of UPR might exist in A549 cells.

Importantly, we uncovered that ANKS4B is a negative regulator of autophagy. Currently, controversy exists among studies on the role of autophagy in ZIKV replication (Ke, 2018). Many studies support that ZIKV utilizes cellular autophagy response for its replication (Ozeki et al., 2001; Hamel et al., 2015; Liang et al., 2016; Ke, 2018; Abernathy et al., 2019) while other studies showed autophagy has no significant effect or an antiviral role (Liu et al., 2018; Ojha et al., 2019). These discrepancies might be due to different infection models used. The ZIKV infection induces autophagy in different cells, including skin fibroblasts (Hamel et al., 2015), fetal neural stem cells (Liang et al., 2016), cytotrophoblasts (Cao et al., 2017), and human umbilical vein endothelial cells (Peng et al., 2018). Present work provided evidence that autophagy also occurred in liver cells (Huh7) upon ZIKV infection, and inhibition of autophagy by CQ or 3-MA downregulates its replication, supporting that autophagy plays a proviral role in Huh7 cells. Interestingly, the ANKS4B depletion led to a higher extent of autophagy response, indicating that ANKS4B inhibits the autophagic process. Considering that ANKS4B is associated with ER membrane, we proposed that ANKS4B might disturb formation of autophagosome through forming protein complex with some ER proteins.

Finally, we established that the antiviral effect of ANKS4B exerts through its regulation on autophagic process. CQ inhibits the replication of ZIKV in HUVEC (Peng et al., 2018), Vero, and MEF cells (Zhang et al., 2019) through inhibiting the endosomal viral RNA release and autophagy. In our study, we treated cells after viral entry, so the inhibition of viral replication by CQ mainly depends on the autophagy. Interestingly, we observed that CQ or 3-MA treatment not only downregulated the viral replication in wild type cells, but also in the ANKS4B-KO cells, indicating that the antiviral action of ANKS4B at least partially relies on its suppression of autophagy.

In summary, our work identified a new restriction factor of ZIKV, ANKS4B, which dampens the autophagic process and hence downregulates the viral multiplication. To the best of our knowledge, role of ANKS4B in virus infection has not been reported before. It is worthwhile to point out, the expression of ANKS4B is gradually downregulated upon ZIKV infection, suggesting that ZIKV might have evolved some antagonism strategy against ANKS4B. Further investigation is warrant to improve our understanding on the interaction between ZIKV and ANKS4B.

## Data Availability Statement

Publicly available datasets were analyzed in this study. This data can be found here: mRNA microarray data have been deposited to the NCBI GEO database (https://www.ncbi.nlm.nih.gov/geo/query/acc.cgi?acc=GSE124094). The accession number is GSE124094.

## Ethics Statement

The animal study was reviewed and approved by the Animal Ethics Committee of Zhongshan School of Medicine, Sun Yat-sen University (ethics reference number: 2020-000211).

## Author Contributions

QL, SZ, and PZ conceived the project. QL and SZ validated the data. QL, SZ, YH, ZH, CC, and XL performed the investigation. QL, SZ, CL, and JH performed the formal analysis, visualization, and original draft preparation. QL and PZ reviewed and edited the manuscript. PZ and CL supervised and administrated the project. PZ, CL, JH, and CC acquired funding. All authors contributed to the article and approved the submitted version.

## Conflict of Interest

The authors declare that the research was conducted in the absence of any commercial or financial relationships that could be construed as a potential conflict of interest.
